# Granulocyte colony-stimulating factor (G-CSF) mediates bone resorption in periodontitis

**DOI:** 10.1186/s12903-021-01658-1

**Published:** 2021-06-12

**Authors:** Hui Yu, Tianyi Zhang, Haibin Lu, Qi Ma, Dong Zhao, Jiang Sun, Zuomin Wang

**Affiliations:** 1 Department of Stomatology, Beijing Chao-Yang Hospital, Capital Medical University, 8th Gongti South Road, Beijing, China; 2 Department of Stomatology, Affiliated Zhongshan Hospital of Dalian University, 6th Jiefang Street, Dalian, Liaoning China; 3 Shanxi Medical University, 382th WuyiRoad, Xinghualing Distrct, Taiyuan, Shanxi China; 4 Department of Pathology, Affiliated Zhongshan Hospital of Dalian University, 6th Jiefang Street, Dalian, Liaoning China; 5 Department of Periodontology, Dalian Stomatological Hospital, 935th Changjiang Road, Dalian, Liaoning China

## Abstract

**Background:**

Granulocyte colony-stimulating factor (G-CSF) is an important immune factor that mediates bone metabolism by regulating the functions of osteoclasts and osteoblasts. Bone loss is a serious and progressive result of periodontitis. However, the mechanisms underlying the effects of G-CSF on periodontal inflammation have yet not been completely elucidated. Here, we examined whether an anti-G-CSF antibody could inhibit bone resorption in a model of experimental periodontitis and investigated the local expression of G-CSF in periodontal tissues.

**Methods:**

Experimental periodontitis was induced in mice using ligatures. The levels of G-CSF in serum and bone marrow were measured; immunofluorescence was then performed to analyze the localization and expression of G-CSF in periodontal tissues. Mice with periodontitis were administered anti-G-CSF antibody by tail vein injection to assess the inhibition of bone resorption. Three-dimensional reconstruction was performed to measure bone destruction‐related parameters via micro-computed tomography analysis. Immunofluorescence staining was used to investigate the presence of osteocalcin-positive osteoblasts; tartrate-resistant acid phosphatase (TRAP) staining was used to observe osteoclast activity in alveolar bone.

**Results:**

The level of G-CSF in serum was significantly elevated in mice with periodontitis. Immunofluorescence analyses showed that G-CSF was mostly expressed in the cell membrane of gingival epithelial cells; this expression was enhanced in the periodontitis group. Additionally, systemic administration of anti-G-CSF antibody significantly inhibited alveolar bone resorption, as evidenced by improvements in bone volume/total volume, bone surface area/bone volume, trabecular thickness, trabecular spacing, and trabecular pattern factor values. Immunofluorescence analysis revealed an enhanced number of osteocalcin-positive osteoblasts, while TRAP staining revealed reduction of osteoclast activity.

**Conclusions:**

G-CSF expression levels were significantly up-regulated in the serum and gingival epithelial cells. Together, anti-G-CSF antibody administration could alleviates alveolar bone resorption, suggesting that G-CSF may be one of the essential immune factors that mediate the bone loss in periodontitis.

## Background

Periodontitis is a bacterial infection-driven inflammatory disease in periodontal tissues. Recently, the Global Burden of Disease study (1990–2010) showed that oral conditions remain a substantial public health problem; globally, 796 million people exhibit severe periodontitis, while 267 million people have total tooth loss. Accordingly, severe periodontitis is the sixth most prevalent disease worldwide [1]. Characteristic features of periodontitis include the formation of periodontal pockets, as well as attachment loss and alveolar bone resorption; these are irreversible and lead to tooth loss. Alveolar bone resorption is the principal indicator of periodontitis progression [2]. Therefore, the prevention of alveolar bone loss and promotion of bone regeneration are important clinical challenges in the treatment of patients with periodontitis. Despite continuous advancements in periodontology techniques (e.g., subgingival scaling, root planing, and laser treatment), the primary goal of periodontal treatment remains the mechanical removal of biofilms. However, it is difficult to recover the lost bone because of an insufficient understanding of the host immune response [3]. The occurrence of periodontitis requires an interaction between the periodontal microbial biofilm and the host immune response, which leads to alveolar bone loss [4]. Although the plaque biofilm contributes to initial onset of periodontitis, its presence is not essential for periodontitis progression [5]. The concept of “bone immunity” has been proposed by some researchers. This concept suggests that the skeletal and immune systems are closely related and interact with each other; immune system abnormalities may therefore cause changes in bone metabolism [6]. This theory provides a new perspective whereby alveolar bone loss can be controlled and prevented by regulating the local immune response to periodontal inflammation [7].

Immune cells are involved in the maintenance of a healthy periodontal equilibrium. Inflamed periodontal tissues produce various proinflammatory cytokines that participate in osteoclastogenesis and collagen degradation in periodontitis; these include interleukins (e.g., IL-1α, IL-1β, and IL-6), tumor necrosis factor-α, matrix metalloproteinases, and granulocyte colony-stimulating factor (G-CSF) [8, 9]. G-CSF is an important immune factor that participates in neutrophil production, mobilization, survival, and chemotaxis [10]. Neutrophils form the primary defense system in periodontal tissues. Notably, a lack of neutrophil monitoring of bacterial infection is considered the cause of excessive periodontal deterioration [2]. There is some evidence that the level of G-CSF is elevated in the peripheral blood of patients with periodontitis [11]. G-CSF is widely used in clinical treatment of patients with congenital agranulocytosis; 28% of those patients develop osteoporosis after treatment with G-CSF [12]. This clinical phenomenon has also been verified in animal experiments; after long-term treatment with G-CSF, diminished bone mass was associated with significant reduction of mature osteoblasts [13]. Additionally, G-CSF has been reported to mediate osteoblast reduction in an animal model of sepsis [14].

In our previous study, we have found that the level of G-CSF in gingival tissues was markedly increased in the periodontitis group, and the higher expression of G-CSF was correlated with higher infiltration of immune cells, especially with neutrophil infiltration [15]. But, the relationship between G-CSF and periodontal bone loss has not been fully elucidated. So, in this study, we mainly focused on whether G-CSF was one of the key inflammatory factors that mediates bone resorption in periodontitis. To determine the roles of G-CSF in periodontal bone loss, we investigated the localization and expression of G-CSF, as well as the effects of anti-G-CSF antibody administration, in a mouse model of experimental periodontitis.

## Methods

### Mice

This study was conducted in accordance with the tenets of the Declaration of Helsinki. All experiments involving studies performed with animals were approved by the Ethics Committee of the Affiliated Zhongshan Hospital of Dalian University (ethics approval number: 2019081503). Eight-week-old specific-pathogen-free C57BL/6 male mice (weight: 20 ± 10 g) were obtained from the Experimental Animal Center of Dalian Medical University (Dalian, China). Male mice were selected because they have better physical health indicators than female mice, which can help to reduce experimental artifacts or problems interpreting the findings. During the experiment, the mice were provided standard laboratory food and water. After the experimental period, the mice were sacrificed following the induction of general anesthesia.

### Establishment of periodontitis model

After the mice had been adapted to the feeding environment for 1 week, a subset of mice was divided into two groups: control (n = 10, without ligature placement) and periodontitis (n = 10). For establishment of the periodontitis model: general anesthesia was induced in the mice using intraperitoneal injections of 0.5% pentobarbital sodium (0.3 ml per mouse). Experimental periodontitis was then induced with a 5–0 silk ligature (SA82G, Mersilk, Ethicon, NJ, USA) around the cervical portion of the maxillary second molar without gingival damage [16]. After 5 weeks of periodontitis modeling, mice were sacrificed to assess the local and systemic expression levels of G-CSF. Another subset of mice was divided into three groups: (1) control (n = 6; each mouse did not undergo ligature placement, but received 1.5 µg of control IgG; MAB005, R&D Systems, Minneapolis, MN, USA); (2) periodontitis (n = 6; each mouse underwent induction of experimental periodontitis, then received 1.5 µg of control IgG); (3) periodontitis + anti-G-CSF (n = 6; each mouse underwent induction of experimental periodontitis, then received 1.5 µg of anti-G-CSF antibody; MAB414, R&D Systems). Antibody administration was performed by tail vein injection at 3-day intervals for 2 weeks. After 5 weeks, the mice were sacrificed to investigate the effects of anti-G-CSF antibody administration on the loss of bone mass.

### Sample collection

Serum extraction: After the induction of anesthesia, 1.5–2 ml blood samples were obtained by the removal of mouse eyeballs. The blood was incubated at room temperature for 2 h to enable full solidification. It was then centrifuged at 3000×*g* for 15 min. The separated serum was quickly transferred to new tubes and stored at -80 °C for subsequent analysis.

Extraction of bone marrow supernatant: After the induction of euthanasia, mice were placed on a super-clean platform and soaked in 75% alcohol for 5 min. One set of surgical instruments was used to separate mouse skin; a second set of surgical instruments was then used to remove the muscle and tendon tissue attached to the femur. Subsequently, the femur and tibia were extracted; the femoral metaphysis was severed and washed with 1 ml of heparinized phosphate-buffered saline to collect bone marrow. The suspension was centrifuged at 3000×*g* for 15 min; the supernatant was then collected and stored at − 80 °C for subsequent analysis.

Maxillary sample fixation: Mouse maxillary specimens were fixed in 4% paraformaldehyde for 48 h, then subjected to micro-computed tomography (Inveon™ Multi Modality; Siemens Medical Solutions, Inc., Malvern, PA, USA) analyses.

Maxillary decalcification: Maxillary specimens were decalcified with ethylene diamine tetraacetic acid (EDTA) disodium salt (E1171, Solarbio, Beijing, China) for 5 weeks. The EDTA decalcifying liquid was changed at 4-day intervals until each sample could be probed without resistance. All samples were then embedded in paraffin wax for subsequent analysis.

### Enzyme linked immunosorbent assay (ELISA)

The levels of G-CSF in mouse serum and bone marrow supernatant were measured using a mouse G-CSF ELISA kit (EM0086, Fine test, Wuhan, China) in accordance with the manufacturer’s protocol. Briefly, a double-antibody sandwich ELISA method was used. Plates were pre-coated with mouse anti-G-CSF antibody. A biotin-conjugated antibody was used as a detection antibody. The standards, test samples, and biotin-conjugated detection antibody were then added to the wells, followed by wash buffer. Horseradish peroxidase (HRP)-streptavidin was added and unbound conjugates were removed with wash buffer. Tetramethylbenzidine (TMB) substrates were used to visualize the HRP enzymatic reaction, such that HRP interacted with TMB to produce a blue color that became yellow after the addition of acidic stop solution. The intensity of the yellow color was proportional to the amount of G-CSF in each sample. Optical density values were measured using a microplate reader (240,351, Bio Tek, Winooski, VT, USA). The absorbance was set at 450 nm.

### Micro-computed tomography scanning and analysis

The computed tomography settings were as follows: voltage, 80 kV; current, 80 µA; number of images, 1024. The three-dimensional images were reconstructed using software provided within the computed tomography device (Inveon™ Image Research Place). After reconstruction, the root bifurcation region in the second molar was set as the region of interest (a column-area volume of 0.0058 mm^3^) and a standardized threshold was used to analyze the bone destruction‐related parameters (i.e., bone volume/total volume, bone surface area/bone volume, trabecular thickness, trabecular number, trabecular spacing, and trabecular pattern factor).

### Histological and immunofluorescence staining

Coronal serial sections (3.5 µm thickness) were affixed to adhesive slides and stained with hematoxylin and eosin. Immunofluorescence staining was performed in accordance with standard protocols. Briefly, conventional dewaxing methods were performed; slides were then incubated in citrate buffer (pH = 6.0, PN5360, G-CLONE, Beijing, China) that was subjected to microwave heating for antigen retrieval. Subsequently, endogenous peroxidases were inactivated by incubation in 3% H_2_O_2_–methanol at room temperature for 10 min. Slides were then blocked with goat serum sealant (KGSP03, Keygen, Jiangsu, China) at room temperature for 20 min. Immunofluorescence staining was performed with a primary antibody against G-CSF (1:50 dilution, ab181053, Abcam, Cambridge, UK) via incubation in a humidified environment for 2 h at 37℃. Secondary antibody detection was performed with goat anti-rabbit fluorescein isothiocyanate (FITC) (1:50 dilution, 111-095-003, Jackson ImmunoResearch, West Grove, PA, USA), via incubation in darkness at 37℃ for 1 h. Vectashield mounting medium with 4′,6-diamidino-2-phenylindole (DAPI) (KGA215, Keygen) was used for nuclear staining (5 min incubation at room temperature). Imaging was performed with a confocal microscope (BX43, Olympus, Tokyo, Japan).

### Immunohistochemistry

Paraffin-embedded sections were dewaxed, hydrated, and heat-treated as described in the previous section. An immunohistochemical staining kit (KGSP03, Keygen) with anti-rabbit IgG by streptavidin-peroxidase was used. First, 3% H_2_O_2_ was used to inactivate endogenous peroxidases at room temperature for 10 min, followed by blocking with goat serum sealant (KGSP03, Keygen) at room temperature for 10 min. Slides were then incubated with a primary antibody against osteocalcin (OCN) (1:100 dilution, 193876, Abcam) overnight at 4 °C. Secondary antibody detection was performed with anti-rabbit biotin (KGSP03, Keygen), via incubation for 10 min at room temperature. Chain-affinity HRP-labeling was then performed via incubation for 10 min at room temperature. Finally, tissue sections were directly exposed to diaminobenzidine (DAB) chromogen (KGSP03, Keygen) and counterstained with hematoxylin for 5 min. For measurement of integral optical density, three random images were captured from each sample using the 40 × objective in a microscope with an attached digital camera (D600, Canon Optical Company, Ltd., Tokyo, Japan). The images were then analyzed with Image-Pro Plus 6.0 software (Media Cybernetics Inc., Bethesda, MD, USA) to quantify the average integral optical density of OCN-positive cells.

### Tartrate-resistant acid phosphatase (TRAP) staining

Sections were stained for TRAP activity using a TRAP assay kit (KGA221, Keygen), in accordance with the manufacturer’s instructions. After staining had been performed, multinucleated osteoclasts (adherent to alveolar bone surfaces) were identified using a light microscope (BX53, Olympus). The numbers of TRAP-positive cells were quantified in three random images captured from each sample using the 40 × objective in a light microscope.

### Statistical analysis

IBM SPSS Statistics, version 22.0 (IBM Corp., Armonk, NY, USA) was used for statistical analysis. All quantitative data are presented as means ± standard deviations. Statistical analyses were performed using one-way analysis of variance. Student’s t-test was used to identify statistically significant differences. Values of *P* < 0.05 were considered statistically significant.

## Results

### Levels of G-CSF in serum and bone marrow

**Fig. 1:**
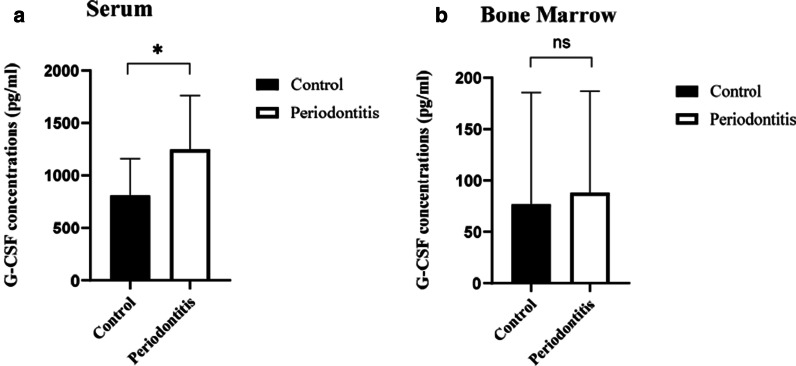
ELISA data showing levels of G-CSF in serum and bone marrow in control and mouse model of periodontitis. A significantly greater level of G-CSF in serum from the periodontitis group, compared with the control group (**A**). The amount of G-CSF in bone marrow tended to be upregulated in the periodontitis group, but there was no difference between the two groups (**B**). Data are presented as mean ± SD. *P < 0.05 using two-tailed Students t-test. ns, no differences

To investigate the levels of G-CSF in our mouse model of periodontitis, we performed ELISA analyses of both serum and bone marrow. Our results showed a significantly greater level of G-CSF in serum from the periodontitis group, compared with the control group (Fig. 1A, *P* < 0.05). The amount of G-CSF in bone marrow tended to be upregulated in the periodontitis group, but this difference was not statistically significant (Fig. 1B, *P* > 0.05).

### Localization and expression of G-CSF in periodontal tissue

**Fig. 2:**
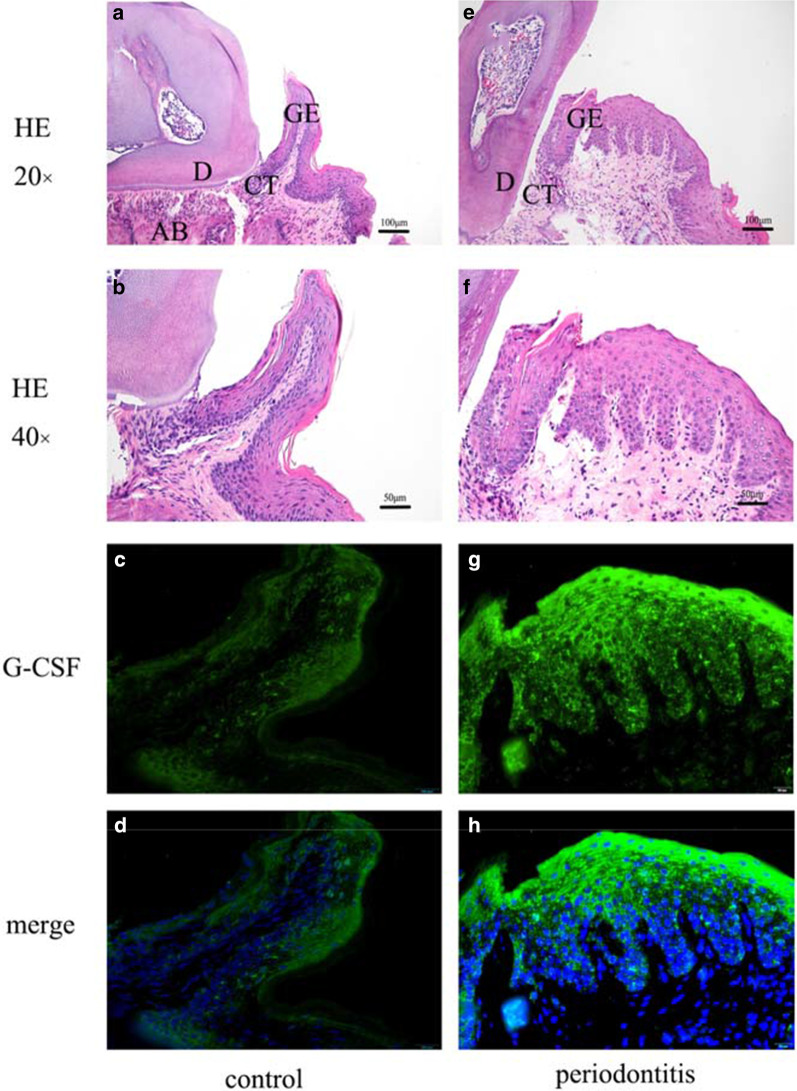
Immunofluorescence localization and expression of G-CSF in control group and mouse model of periodontitis. Images of the gingival junctional epithelium were taken by hematoxylin–eosin staining (**A**, **B**, **E**, **F**) with 20 × and 40 × objectives. The membranes of positive cells exhibited green fluorescence (**C**, **G**), while the nuclei exhibited blue fluorescence (**D**, **H**). Control group (**A** to **D**), periodontitis group (**E** to **H**), merged images (**D**, **H**). **C**, **D**, **G** and **H**: Bars, 20um. D, dentin; GE, gingival epithelium; CT, connective tissue; AB, alveolar bone

To evaluate changes in G-CSF localization in periodontal tissue our mouse model of periodontitis, we performed immunofluorescence analysis (Fig. 2). Images of the gingival junctional epithelium were taken by hematoxylin–eosin staining (Fig. 2A, B, E, F) with 20 × and 40 × objectives, using a light microscope. The results indicated that G-CSF was localized in the membranes of gingival epithelial cells in control mice (Fig. 2C). The membranes of positive cells exhibited green fluorescence (Fig. 2C, G), while the nuclei exhibited blue fluorescence (Fig. 2D, H). The membrane immunoreactivity of G-CSF was enhanced in the periodontitis group (Fig. 2H), compared with the control group (Fig. 2D).

### Effects of anti-G-CSF antibody administration on alveolar bone loss

**Fig. 3:**
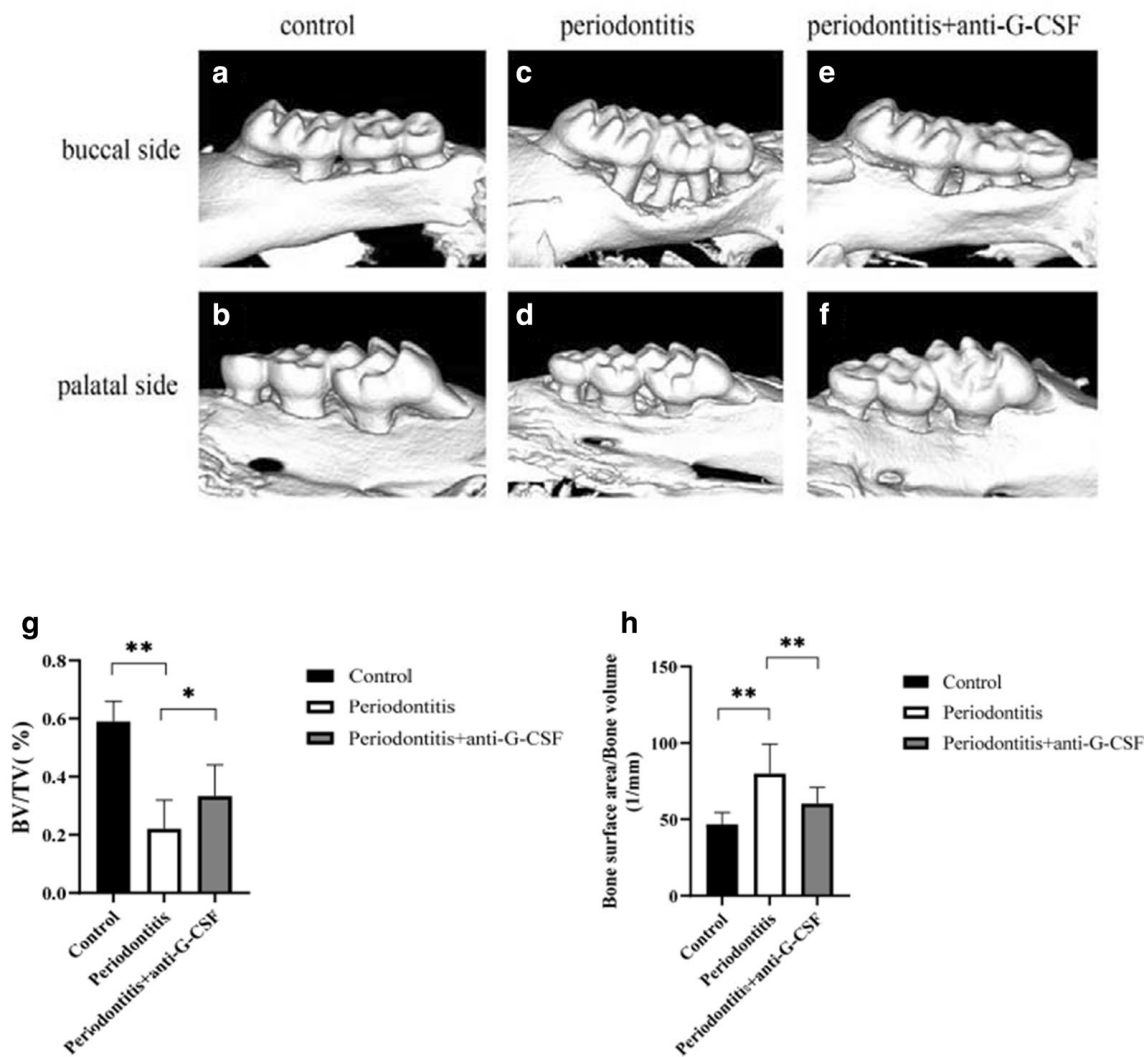

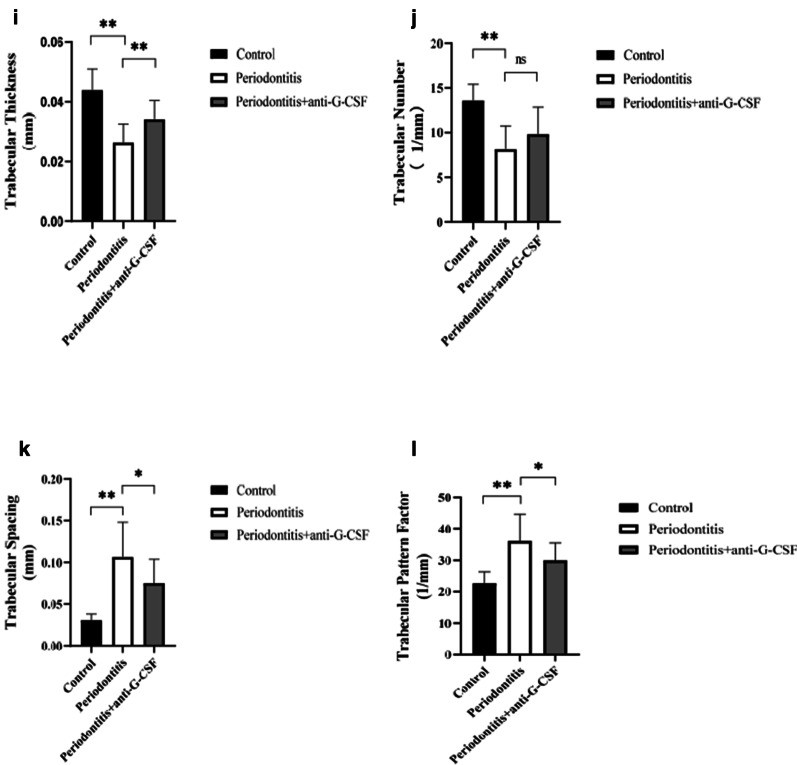
The three‐dimensional images of micro-computed tomography reconstructed. We captured three-dimensional images of buccal sides (**A**, **C**, **E**) and palatal sides (**B**, **D**, **F**) of periodontal tissues. The bone volumes of mice in the periodontitis group (**C**, **D**) were markedly reduced, compared with those of mice in the control group (**A**, **B**). Notably, mice in the periodontitis + anti-G-CSF group showed significant attenuation of alveolar bone resorption (**E**, **F**), bone mass loss was also partially alleviated. Mice in the periodontitis + anti-G-CSF group also showed improvements in bone volume/total volume (**G**), bone surface area/bone volume (**H**), trabecular thickness (**I**), trabecular spacing (**K**), and trabecular pattern factor (**L**) values, compared with mice in the periodontitis group (*P* < 0.05). However, there were no differences in trabecular number values (**J**, *P* > 0.05). Data are shown as mean ± SD, *P < 0.05, **P < 0.01; ns, no differences. One-way ANOVA with Bonferroni correction

To examine whether anti-G-CSF antibody administration could inhibit bone resorption, we performed micro-computed tomography analysis of bone-related parameters. We captured three-dimensional images of the buccal sides (Fig. 3A, C, E) and palatal sides (Fig. 3B, D, F) of periodontal tissues. Quantitation of these three-dimensional images indicated that the bone volumes of mice in the periodontitis group (Fig. 3C, D) were markedly reduced, compared with those of mice in the control group (Fig. 3A, B). Notably, mice in the periodontitis + anti-G-CSF group showed significant attenuation of alveolar bone resorption (Fig. 3E, F); bone mass loss was also partially alleviated. Mice in the periodontitis + anti-G-CSF group also showed improvements in bone volume/total volume (Fig. 3G), bone surface area/bone volume (Fig. 3H), trabecular thickness (Fig. 3I), trabecular spacing (Fig. 3K), and trabecular pattern factor (Fig. 3L) values, compared with mice in the periodontitis group (*P* < 0.05). However, there were no differences in trabecular number values (Fig. 3J, *P* > 0.05).

### Effects of anti-G-CSF antibody administration on osteogenic ability

**Fig. 4:**
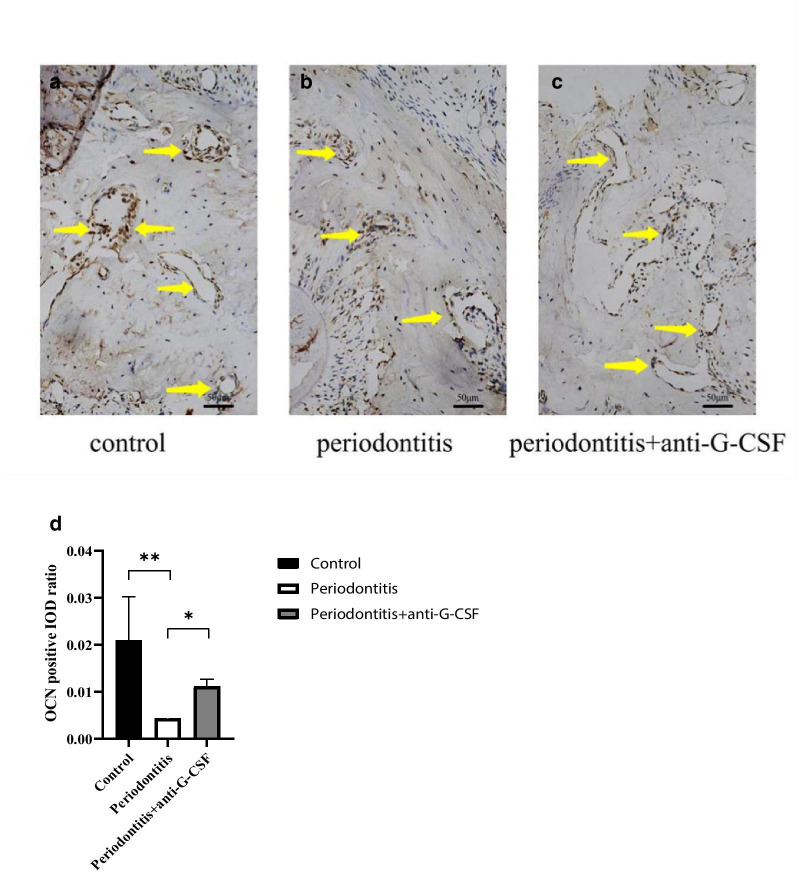
Quantification of osteoblasts and representative images of immunohistochemical staining (**A** to **C**). The periodontitis + anti-G-CSF group demonstrated an enhanced number of osteoblasts, compared with the periodontitis group (**D**). The yellow arrow indicates osteoblasts. Data are presented as mean ± SD. *P < 0.05, **P < 0.01; One-way ANOVA with Bonferroni correction

OCN is synthesized and secreted by osteoblasts; thus, it comprises a specific indicator of bone regeneration and bone formation [6]. Immunohistochemical staining was performed to analyze the presence of OCN-positive osteoblasts. As shown in Fig. 4A–C, the number of OCN-positive osteoblasts was lower in the periodontitis group (Fig. 4B) than in the periodontitis + anti-G-CSF group (Fig. 4C). The periodontitis + anti-G-CSF group demonstrated an enhanced number of osteoblasts, compared with the periodontitis group (Fig. 4D, *P* < 0.05).

### Effects of anti-G-CSF antibody administration on osteoclasticity

**Fig. 5:**
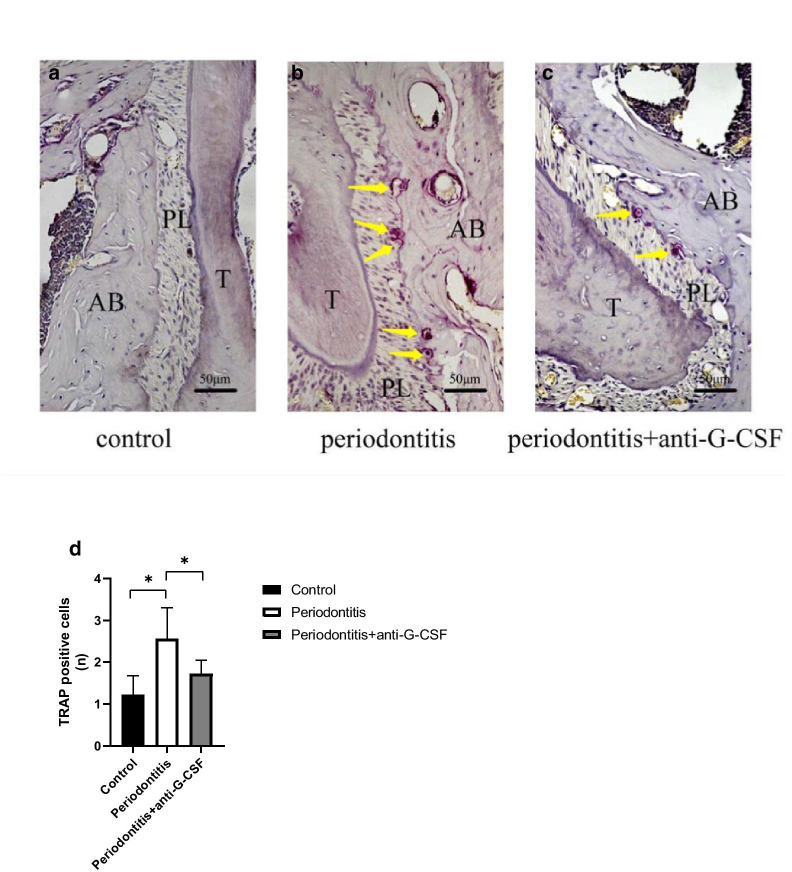
TRAP staining was used to detect the osteoclasts (**A** to **C**). TRAP-positive osteoclasts (red color) were arrayed along alveolar bone surface. Quantitative analysis was performed on the number of TRAP-positive osteoclasts (**D**). The periodontitis + anti-G-CSF group exhibited a significantly reduced number of TRAP-positive osteoclasts, compared with the periodontitis group. Yellow arrow indicates TRAP-positive osteoclasts. AB: alveolar bone, T: tooth, PL: periodontal ligament. Data are presented as mean ± SD. *P < 0.05, using one-way ANOVA with Bonferroni correction

Bone mass can be diminished because of reduced osteogenic capacity, as well as enhanced osteoclast capacity [2]. TRAP is mainly secreted by activated osteoclasts. Here, we conducted TRAP staining to assess changes in osteoclast capacity and bone resorption. Our findings revealed that, compared with the control group (Fig. 5A), bone resorption was significantly worse in the periodontitis group (Fig. 5B); moreover, the periodontitis group exhibited many TRAP-positive multinucleated osteoclasts along the margin of alveolar bone resorption (Fig. 5B). Notably, the periodontitis + anti-G-CSF group (Fig. 5C) exhibited a significantly reduced number of TRAP-positive osteoclasts, compared with the periodontitis group (Fig. 5D, *P* < 0.05).

## Discussion

Alveolar bone is the most active site of bone metabolism and remodeling in the human skeletal system. The most serious damage caused by periodontitis is tooth resorption or loss because of periodontal tissue destruction. Research concerning periodontitis pathogenesis has revealed that plaque biofilm is an initial risk factor for periodontitis; however, some periodontitis-related tissue destruction is caused by the host immune response to the bacteria and toxic factors within the biofilm [4]. Neutrophils form the first defense against bacteria in periodontal tissues. The migration of neutrophils through the junctional epithelium into the gingival sulcus is a continuous process, such that transmigration is a hallmark of infection [17]. Moreover, periodontitis is a local manifestation of systemic immunoreactivity. Mature neutrophils are stimulated by bacteria and their products such as lipopolysaccharide; they then infiltrate sites of inflammation and phagocytize bacteria under the guidance of cytokines, adhesion molecules, and chemokines. Neutrophils also contribute to periodontal inflammation [17]. Excessive neutrophil reactions to pathogens can cause immune damage. G-CSF is commonly found in multiple tissues and plays a vital role in the immune system [18]. G-CSF is a critical mediator of neutrophil release from the bone marrow, which induces neutrophil mobilization, proliferation, and differentiation [19]; some studies have shown that the level of G-CSF is elevated in peripheral blood from patients with periodontitis [11], but an opposite relationship has been demonstrated in other studies [20]. Furthermore, some adolescent patients with systemic lupus erythematosus have poor periodontal statuses, with higher levels of G-CSF in gingival crevicular fluid and serum [21]. In our previous study, the level of G-CSF in gingival tissues was markedly increased in the periodontitis group [15]. In the present study, we found that the level of G-CSF in serum was significantly higher in mice with experimental periodontitis, compared with the control group (Fig. 1A). Also, the level of G-CSF in bone marrow tended to be upregulated, but this difference was not statistically significant (Fig. 1B). Immunofluorescence analyses further demonstrated the localization and expression of G-CSF. Notably, G-CSF was strongly expressed in the membranes of gingival epithelial cells in mice in the periodontitis group (Fig. 2H), compared with mice in the control group (Fig. 2D). Taken together with the previous studies, our findings suggest that G-CSF is a critical immune factor that contributes to the onset of periodontitis.

In the context of periodontitis, the decline in osteoblast function partly occurs because of osteoblast apoptosis, which is mediated by the secretion of G-CSF from bone marrow nucleated cells [22]. *Staphylococcus aureus* infection has been shown to suppress the osteoblastic cell function and induce progressive bone loss by upregulating the level of G-CSF [23]. Furthermore, G-CSF inhibits the growth of both osteoblasts and osteocytes by upregulating the production of nitric oxide from neutrophils [24]. There were some evidences that G-CSF affects osteoblasts. To the best of our knowledge, there have been few studies concerning the effects of G-CSF on alveolar bone resorption and bone loss in the context of periodontitis. To precisely examine whether the administration of an anti-G-CSF antibody could inhibit bone resorption, we used micro-computed tomography to evaluate the irregular bone loss in the second molar root bifurcation region in our mouse model of periodontitis. The bone volumes of mice in the periodontitis group were markedly reduced (Fig. 3C, D), compared with those of mice in the control group (Fig. 3A, B). Furthermore, bone resorption was significantly inhibited by anti-G-CSF antibody administration (Fig. 3E, F). Our results implied that G-CSF blockage could alleviate alveolar bone resorption and height reduction.

The maintenance of normal bone metabolism includes both formation and destruction of bone. Changes in bone mass are regulated by osteogenic and osteoclastic characteristics. A study have revealed that in G-CSF induced hematopoietic stem progenitor cells mobilization, the expression of osteoblasts and OCN were significantly reduced [25]. Moreover, human osteoblasts treated with recombinant human G-CSF showed slightly lower rates of proliferation [26]. Furthermore, in humans, serum osteocalcin concentration, a specific marker of bone formation, was strongly reduced after 3 days of G-CSF administration [27]. The expression of Runx2, a transcription factor controlling osteoblast function, was dramatically downregulated by G-CSF administration in the bone marrow of mice [28]. OCN is synthesized and secreted by osteoblasts; thus, it comprises a specific indicator of bone regeneration and bone formation. Here, we conducted immunohistochemistry to evaluate changes in OCN during osteogenesis. Our findings are consistent with those of prior studies [13, 14], such that the number of OCN-positive osteoblasts was consistently lower among mice in the periodontitis group (Fig. 4B) than among mice in the periodontitis + anti-G-CSF group (Fig. 4C). These results demonstrated that anti-G-CSF antibody treatment led to improved osteoblast activity and enhanced bone formation. Furthermore, our findings suggest that G-CSF may constitute a crucial immune factor that participates in alveolar bone resorption during periodontitis.

In vitro analyses of human cells have shown that G-CSF can reduce the number and activity of pre-osteoblasts through B-lymphocyte-mediated signaling; G-CSF can also inhibit osteoclast activity through T-lymphocyte-mediated signaling [29]. Notably, the short-term use of G-CSF in children with cancer can affect bone metabolism and contribute to metabolic changes. Specifically, reduced osteoblastic activity and enhanced osteoclastic activity may lead to osteoporosis-related bone pain in these patients [30]. Osteoblasts promote the formation of new bone, while osteoclasts promote bone resorption; these opposing effects can maintain bone remodeling balance. Additionally, osteoclasts are dispensable for hematopoietic progenitor mobilization by G-CSF in mice [31]; short-term G-CSF treatment leads to reduced osteoblast number and osteoclast stimulation [32]. In this study, we used TRAP staining to assess changes in osteoclast capacity and bone resorption in our mouse model of periodontitis. TRAP is mainly secreted by activated osteoclasts. Importantly, we found that the number of TRAP-positive osteoclasts was significantly reduced in the periodontitis + anti-G-CSF group (Fig. 5C). These results suggested that anti-G-CSF antibody treatment could reduce osteoclast activity and alleviate alveolar bone absorption.

Our results suggested that G-CSF may be an important inflammatory factor that mediates periodontitis-related bone loss, although the specific mechanism remains unclear. In a future study, we will perform a detailed investigation of the cellular mechanism of G-CSF-mediated bone loss.

## Conclusions

In conclusion, we found that G-CSF was highly expressed in periodontal tissues in a mouse model of periodontitis. Anti-G-CSF antibody treatment significantly inhibited alveolar bone resorption, as evidenced by improvements in bone volume/total volume, bone surface area/bone volume, trabecular thickness, trabecular spacing, and trabecular pattern factor values, as well as by changes in the numbers of cells that exhibited expression of osteocalcin and TRAP. These findings indicate that G-CSF may be one of the essential immune factors that mediate the bone loss in periodontitis. Moreover, the findings constitute new evidence to support novel immune-mediated treatments for bone damage in the context of periodontitis.

## Data Availability

All raw data used and analyzed during the current research are available from the corresponding author on reasonable request.

## Abbreviations

G-CSF: Granulocyte colony stimulating factor
TRAP: Tartrate-resistant acidic phosphataseincluding
EDTA: Ethylene diamine tetraacetic acid
ELISA: Enzyme linked immunosorbent assay
HRP: Horseradish peroxidase
TMB: Tetramethylbenzidine
FITC: Fluorescein isothiocyanate
DAPI: 4′, 6-diamidino-2-phenylindole
OCN: Osteocalcin
DAB: 3,3′-diaminobenzidine
SD: Standard deviations
